# DNA Extraction Method Affects the Detection of a Fungal Pathogen in Formalin-Fixed Specimens Using qPCR

**DOI:** 10.1371/journal.pone.0135389

**Published:** 2015-08-20

**Authors:** Andrea J. Adams, John P. LaBonte, Morgan L. Ball, Kathryn L. Richards-Hrdlicka, Mary H. Toothman, Cheryl J. Briggs

**Affiliations:** 1 Department of Ecology, Evolution, and Marine Biology, University of California, Santa Barbara, California, United States of America; 2 Wildlands Conservation Science, Lompoc, California, United States of America; 3 School of Forestry and Environmental Studies, Yale University, New Haven, Connecticut, United States of America; University of South Dakota, UNITED STATES

## Abstract

Museum collections provide indispensable repositories for obtaining information about the historical presence of disease in wildlife populations. The pathogenic amphibian chytrid fungus *Batrachochytrium dendrobatidis* (Bd) has played a significant role in global amphibian declines, and examining preserved specimens for Bd can improve our understanding of its emergence and spread. Quantitative PCR (qPCR) enables Bd detection with minimal disturbance to amphibian skin and is significantly more sensitive to detecting Bd than histology; therefore, developing effective qPCR methodologies for detecting Bd DNA in formalin-fixed specimens can provide an efficient and effective approach to examining historical Bd emergence and prevalence. Techniques for detecting Bd in museum specimens have not been evaluated for their effectiveness in control specimens that mimic the conditions of animals most likely to be encountered in museums, including those with low pathogen loads. We used American bullfrogs (*Lithobates catesbeianus*) of known infection status to evaluate the success of qPCR to detect Bd in formalin-fixed specimens after three years of ethanol storage. Our objectives were to compare the most commonly used DNA extraction method for Bd (PrepMan, PM) to Macherey-Nagel DNA FFPE (MN), test optimizations for Bd detection with PM, and provide recommendations for maximizing Bd detection. We found that successful detection is relatively high (80–90%) when Bd loads before formalin fixation are high, regardless of the extraction method used; however, at lower infection levels, detection probabilities were significantly reduced. The MN DNA extraction method increased Bd detection by as much as 50% at moderate infection levels. Our results indicate that, for animals characterized by lower pathogen loads (i.e., those most commonly encountered in museum collections), current methods may underestimate the proportion of Bd-infected amphibians. Those extracting DNA from archived museum specimens should ensure that the techniques they are using are known to provide high-quality throughput DNA for later analysis.

## Introduction

Natural history collections are becoming increasingly important for ecological and conservation research [1, 2], facilitating studies as diverse as those documenting the effects of environmental contaminants [3] and morphological responses to anthropogenic climate change [4]. With the advancement of molecular techniques, museum specimens have considerable value for inquiries about infectious diseases and their conservation consequences [5–8]. The discovery and description of the fungal pathogen *Batrachochytrium dendrobatidis* (hereafter Bd) in the late 1990s [9, 10] provided an explanation for many amphibian declines that had previously been enigmatic (e.g., [9, 11, 12]); however, most threatened species have inadequate data tying their declines to Bd as a primary factor [13]. The comprehensive extent of the global distribution of Bd [14] remains unclear, and understanding the spatiotemporal dynamics of Bd emergence is important in evaluating the possible role of this pathogen in the declines of amphibian species.

The “spreading pathogen” hypothesis [15] posits that Bd is a novel pathogen that has recently dispersed around the world. This hypothesis has been the focus of much recent research (as reviewed in [16]) and has been supported by both genetic [17–20] and spatiotemporal data [21, 22]. Museum specimen collections have an increasingly important function in enabling ecologists to address specific questions about the potential role of Bd in historical amphibian populations [2]. Patterns of Bd emergence and spread have been deduced from work with archived amphibian specimens [7, 23–30], using both histological and polymerase chain reaction (PCR) techniques.

Histopathology has been used successfully to identify chytridiomycosis in live and fresh-dead amphibians [9, 10, 31] and in formalin-fixed specimens [7, 23, 25, 32, 33], made possible by the characteristic thickening of metamorphosed amphibian skin, accompanied by reproductive zoosporangia structures presented by cutaneous chytridiomycosis [9, 10, 34, 35]. Histological methods for large-scale sampling, however, are labor intensive and time-consuming, causing issues of feasibility when large numbers of specimens are to be sampled to determine landscape-level effects and emergence of Bd. This can result in small sample sizes, increasing the risk of sampling bias [25, 36]. Infection with Bd typically has a patchy distribution on amphibian skin, and histology can produce false negatives even when Bd’s reproductive zoosporangia are present, if zoosporangia density is low, or if distribution is uneven, which is possible even in highly infected individuals [10, 37–39]. In addition, histological methods require that a portion of the skin be removed for analysis, which damages specimens—a primary concern for museum curators.

The development of PCR [40] and quantitative PCR (qPCR) techniques for detecting Bd [39] has enabled Bd detection with minimal disturbance to amphibian skin and substantially increased sensitivity: qPCR is significantly more sensitive than histology [41, 42]. Because of the limitations posed by histology and the advantages of qPCR, developing effective qPCR-based methods for detecting Bd DNA in formalin-fixed specimens can provide an efficient and effective approach to examining historical Bd emergence and prevalence.

Before 2011, attempts to detect Bd DNA from formalin-fixed specimens were relatively unsuccessful (reviewed in Richards-Hrdlicka [43]). Cheng et al. [24] were able to successfully recover Bd in 83% of histologically-confirmed, infected specimens using PrepMan Ultra DNA extraction reagent (Life Technologies, Grand Island, NY, USA; hereafter PM) and qPCR, following the qPCR protocol of Boyle et al. [39]. This was the first study to effectively use qPCR to detect Bd in infected specimens that had been fixed in formalin, and created a hopeful prospect for applying the same method to other collections. Richards-Hrdlicka [43] also successfully used qPCR to detect Bd from formalin-fixed amphibians using two extraction kits specifically designed to extract DNA from formalin-fixed tissue: DNA IQ (Promega, Madison, WI, USA), and Macherey-Nagel DNA FFPE (Bethlehem, PA, USA; hereafter MN). In other taxa, the MN extraction method has been shown to increase DNA yield and quality as compared to other DNA extraction kits [44]. Since Cheng et al.’s pioneering success with the PM DNA extraction for detecting Bd from formalin-fixed specimens [24, 39], the PM DNA extraction has become the most commonly used method to detect Bd in formalin-fixed specimens [30, 45–50], (although some have used other methods, such as Qiagen spin column-based extractions [24, 47, 49]).

The ability to detect Bd DNA using qPCR can be moderated by several factors, including individual pathogen load. The infection intensity, or Bd load, on an amphibian is commonly measured by taking a skin swab following a standard swabbing protocol [42]. Using qPCR analysis, infection intensity is determined in terms of zoospore equivalents (ZE), the number of zoospores on the swab sample as compared to a standard curve of serial dilutions of standard Bd DNA. Bd loads higher than 10,000 ZE are within the lethal range of some species [22, 51, 52], and specimens with similarly high infection levels have been used to calibrate detection in formalin-fixed tissues [24]. Although it is important to understand detection success in formalin-fixed specimens under high Bd DNA conditions, the most common conditions of museum specimens are likely to be those characterized by low to moderate Bd infection intensities. Low Bd loads are usually exhibited by individuals that are not considered susceptible, are subclinical, are potentially in a post-dieoff, enzootic state (e.g., a median of ≈ 20 ZE was measured in one study of Bd in the enzootic state [52]), or are exhibiting nascent lethal infections. The probability of a collection event coinciding with a dieoff event is small (unless individuals were being collected intentionally because of a dieoff event). Even in multi-year field studies with evidence of Bd-related dieoff events, moribund individuals are rarely encountered after thousands of visual survey hours [53]. In Bd-susceptible species, when individuals reach lethally high Bd loads, they die promptly and morbid amphibians are typically removed quickly by predators or scavengers [54], so those individuals are less likely to be encountered nor desired by collectors. Thus high pathogen loads are likely rare in most herpetological museum collections.

In addition to the importance of understanding Bd detection success under a variety of infection loads, both length of time in formalin and the pH of the solution can affect the ability to detect DNA after fixation of a specimen in formalin [55]. Despite this, the length of time amphibians are exposed to formalin fixative is highly variable. A standard manual for amphibian preservation recommends that amphibians be left in a formalin bath “for a minimum of 4 days, or preferably for the remainder of the field season”[56]; another widely-used source recommends “1 week to 10 days” for formalin fixation [57]. Collectors throughout history have practiced a range of time courses for formalin fixation of amphibian tissues, and many specimens have likely been fixed and/or stored in formalin for longer periods than recommended.

Here, we used qPCR to characterize Bd loads on live frogs then fixed them in formalin, preserved them in ethanol, and re-swabbed them three years later to assess the ability to successfully detect Bd on frogs of known infection status using qPCR. We also compared the efficacy of the PM [24, 39] and MN [43] extraction protocols across a range of zoospore loads. Our specific goals in this study were to: 1) test various optimizations for improving detection of Bd DNA from the commonly-used PM DNA extraction; 2) determine the robustness of qPCR detection of Bd from formalin-fixed museum specimens in conditions that most closely mimic those of formalin-fixed, archived museum specimens; 3) within the aforementioned conditions, examine the effectiveness of PM vs. MN; and 4) determine the potential for false positives to occur in specimen sampling after mixing individuals of known infection status in common containers for formalin fixation and ethanol storage. The results will serve to improve methods used in qPCR detection of Bd DNA from formalin-fixed and ethanol-archived natural history collections.

## Materials and Methods

### Ethics Statement

All specimens were collected in adherence with protocols approved by the University of California, Santa Barbara Institutional Animal Care and Use Committee (266) and permits issued by the California Department of Fish and Wildlife (SC-4436). The non-native American bullfrog (*Lithobates catesbeianus*) is not a legally protected species in the state of California.

### Field Sampling and Preservation

We used the Bd infection status of live frogs to assess our ability to detect Bd DNA after formalin fixation and ethanol preservation. We sampled American bullfrogs (n = 62) for Bd in the wild (Vandenberg Air Force Base, Santa Barbara County, California, USA) using sterile dacron swabs (MW100 fine-tip; Medical Wire & Equipment, Corsham, Wiltshire, England) following Hyatt et al. [42], with five strokes of the swab in each of the six body regions (inner thighs, feet and drink patch; 30 strokes total). Following Boyle et al. [39] to determine Bd loads, we used PM to extract DNA from those swabs [39], and diluted extracts 1:10 in DNase-free 0.25X TE (Tris-EDTA) (hereafter TE). We used triplicate positive controls in quantities of 0.1, 1, 10, and 100 ZE, and triplicate negative controls to detect any false positives. DNA standards were provided by the Australian Animal Health Laboratory, CSIRO Livestock Industries, Victoria, Australia (isolate AAHL 98 1810/3, from Australia), or developed by MHT (isolate CJB7, from California). Standards developed by MHT were quality controlled for equivalent standard quantification to isolate AAHL 98 1810/3 prior to use in qPCR using a protocol provided by the Hyatt laboratory. The Hyatt standard Bd DNA protocol was followed precisely, and new standards were run concurrently with Hyatt standards. Care was taken to ensure a dilution of new standard Bd DNA to 100 ZE (the highest standard DNA amount in the standard curve) matched an average of multi-year Hyatt 100 ZE standard Bd DNA qPCR data, averaged across all plates set to a threshold of 0.1. qPCR amplification parameters followed Boyle et al. [39] and were performed on an Applied Biosystems StepOnePlus Real-Time qPCR System. To calculate a ZE score for each swab, we multiplied raw genomic qPCR output for each sample that was diluted 1:10 by 80 to account for dilution of the sample during the extraction process. Of the 62 bullfrogs sampled in the wild, 94% (n = 58) were Bd-positive at the time of capture, with Bd loads ranging from <1 to over 70,000 ZE (median 17 ZE). The remaining four individuals served as negative controls to test for cross-contamination from storage in common containers.

Because Bd loads detectable on moribund frogs are the most accurate within 24 hours of death [58], bullfrogs were immediately euthanized after swabbing and then fixed in a 10% buffered formalin solution (pH 7) for four days. Since formalin fixation was historically conducted in the field, as trips often lasted for multiple days, immediate formalin fixation is also consistent with methods most likely encountered by those using animals preserved under historical museum specimen methods. After four days of formalin fixation, frogs were placed in a water bath for 20 minutes to rinse them, placed in 70% ethanol for long-term storage, and were assigned randomly to three separate 19 L buckets so that individuals of varying Bd loads were intermixed in the ethanol storage solution, mimicking natural history collections. Specimens were resampled after three years of storage in the ethanol solution.

### Specimen Sampling

To assess Bd detection after formalin fixation and ethanol storage, frogs were re-sampled multiple times for qPCR. We used five separate swabbing events to test the following optimizations, as outlined in Table 1: 1) number of times swabbed (swab events A-C); 2) extract dilution (swab event D); 3) Genereleaser treatment (swab events C & D); 4) TE rehydration (swab events A-C) and 5) DNA extraction method (swab events D & E). Prior to sampling, individual frogs were thoroughly rinsed with clean 70% ethanol to minimize the chances of Bd cross-contamination from other frogs in the same container. Fresh nitrile gloves were used to handle each specimen. Specimens were swabbed in each of the same six areas as live swabbed frogs [42] (bottoms of the feet, inner thighs, drink patch) in five separate events, using a new swab for each individual: first, five times in each of the six areas (5x6; Swab Event “A” in Table 1), followed by 15 strokes in each of the six areas (15x6; “B”, Table 1) and finally 25x6, which was repeated three times (“C”, “D”, “E”, Table 1) to test different extraction techniques. Swab events were discrete and consecutive, so Swab Event B always followed Swab Event A, and so on. We limited the swabs to 25x6 strokes because after 25 strokes in each area, the rayon swab tip began to disintegrate. Even with this many strokes, the specimens remained undamaged. Because no differences have been observed in the probability of Bd detection between the use of swabs and brushes [24], swabs, rather than interdental brushes, were used. These swabs are also less expensive than brushes and identical to those used for field sampling. All specimen swabs were air dried under a laminar flow hood in the laboratory for 48 hours to evaporate residual ethanol from samples prior to DNA extraction.

**Table 1:** Treatments, replicate numbers, and optimizations for each specimen swabbing event, chronological from left to right.

	Specimen swabbing event				
	A	B	C	D	E
**Swab sequence**	5x6	15x6	25x6	25x6	25x6
**Total swab strokes**	30	90	150	150	150
**DNA extraction**	PM	PM	PM	PM	MN
**TE rehydration**	Yes	Yes	Yes	No	No
**Dilution & replicate #**	1:10: singlicate^a^	1:10: singlicate^a^	1:10: singlicate^a^	1:10: duplicate^a^	No dilution; duplicate^a^
**Additional dilution & replicate #**	-	-	-	1:5: singlicate^b^	-
**Additional treatment & replicate #**	-	-	GR singlicate^c^	GR singlicate^d^	-

PM = PrepMan; MN = Macherey-Nagel DNA FFPE; GR = Genereleaser

^a^n = 62;

^b^n = 47;

^c^n = 50

^d^n = 48

### Specimen DNA extractions

To compare the efficacy of subsequent Bd DNA detection using qPCR, DNA from individual swabs were extracted by two separate extraction methods, PM and MN. Although phenol:chloroform may be the extraction method of choice in museum specimens when full animal tissues are being sampled, phenol:chloroform is not demonstrated to be superior when extracting low copy number DNA (e.g., Bd DNA) in samples with a high DNA background (in this case, preserved amphibian DNA) [59], so it was not tested in this study. Multiple optimizations (TE rehydration, reduced dilution, and Genereleaser) were used on PM-extracted swabs in an attempt to improve Bd DNA detection from swabs that were extracted using that method. We performed our pre-PCR sample manipulations (drying, extraction, qPCR plate setup) in a dedicated room separate from our qPCR facilities. This standard work flow for PCR work ensures that unamplified samples cannot be contaminated by post-PCR amplicons. In addition, we followed time-tested decontamination procedures for all benches and equipment (treatment with bleach followed by a 70% ethanol rinse) and employed frequent glove changes to ensure no incidental contamination between samples occurred during pre-PCR processing.

#### PM extraction and optimizations

In the first three PM extractions (Events A-C, Table 1), 20μL TE was added in addition to the PM, to rehydrate the swab in the extraction process and to putatively increase DNA yield from the swab, as TE solubilizes DNA. Swabs from Events A-D (Table 1) were extracted using PM and following Boyle et al. [39], in the same manner described above for the field-collected samples. All PM-extracted swabs (Events A-D) were initially diluted 1:10 with TE [39]. To test if an increase in the amount of DNA template in each reaction would increase recovery rates, additional extract from swabs C and D were diluted only 1:5. To calculate a ZE score for each swab, raw genomic qPCR output for each sample that was diluted 1:10 was multiplied by 80 to account for dilution of the sample during the extraction process. PM-extracted samples that were diluted 1:5 were multiplied by 40. A subset of remaining extracts from swabs C and D were treated with 45 μL of Genereleaser (BioVentures, Murfreesboro, TN, USA), a proprietary formula that quickly releases genetic material from cells and separates inhibitors from the sample, in order to test whether the treatment would increase DNA amplification. All PM-extracted samples were run in singlicate, with the exception of the 1:10 dilution of the D swabs, which were run in duplicate (Table 1).

#### MN Extraction and optimizations

Swabs (Event E) were extracted with MN following Richards-Hrdlicka [43], with minor modifications for the use of swabs rather than interdental brushes. Briefly, 100 μL of FL buffer and 10μL of proteinase K (10mg/mL) were added to the swab and incubated at 37°C overnight for lysis, then 100 μL of D-link crosslink buffer was added and swabs were incubated for 30 minutes at 90°C. After cooling, conditions were adjusted by adding 200 μL of 98% ethanol. To bind the DNA, swab extract was aliquotted into spin columns (TissueSpin XS, Macherey-Nagel) and centrifuged for 1.5 minutes at 2000 rpm, twice, and the flow-through discarded. In some instances, rayon from the swab prevented filter flow-through, so after the first centrifugation, if all of the liquid did not pass through, the filter would be gently agitated with a clean 10μL pipette tip and centrifugation would be repeated. To wash the membrane, 200 μL of B5 buffer was added to the spin column for 5 minutes, then spin columns were centrifuged for 11,000 rpm for 30 seconds, and flow through was discarded. Columns were then placed in 1.5 ml microcentrifuge tubes and left open for 30 minutes to allow ethanol evaporation. To elute the DNA from the filter, BE buffer was warmed to 70°C and 45μL was pipetted directly onto the column’s filter. After 1 minute, columns were centrifuged at 11,000 rpm for 30 seconds. Extracts (5 μL per reaction) were run in duplicate using qPCR as described in Boyle et al. [39]. Bd standards used were the same as described above for field-collected swabs. For all MN extractions, raw genomic output was multiplied by 9 to calculate the total number of ZE per swab (since the total extract volume was 45 μL).

### Data Analysis

A sample was considered positive when any replicates in the sample exhibited a logarithmic curve in the amplification profile that crossed the ΔRn threshold (set at 0.1 [39]). When sample size varied between treatments (refer to superscripts in Table 1), individual swabs were excluded so that those used for paired analyses were from the same frog. Also for paired analyses, when replicate number varied between treatments (singlicate versus duplicate), the first replicate of duplicate runs was used. When comparing PM to MN, the results of duplicate runs were averaged to determine a mean ZE for each sample. We conducted paired t tests (on square root-transformed ZE data to achieve normality) to look for treatment effects on all nonzero ZE values (samples that returned a positive result) and McNemar’s tests to look for treatment effects on recovery rates (number of positives detected/total true positives) within each swab type (C, D, and E).

We used generalized linear mixed models (GLMMs) with a binomial response and a logit-link function to test whether pre-preservation Bd load, DNA extraction method (PM or MN), and individual live frog mass were significant predictors of Bd detection (success or failure) after formalin fixation using duplicate results from swab events D and E. For this analysis, we used only data for individual frogs that we had individual mass recorded (n = 52). We square root-transformed both the live ZE and post-preservation ZE data to facilitate data analysis. We treated initial Bd load, individual mass, and extraction protocol as fixed effects and the identity of each individual frog as a random effect. Treating individual frogs as a random effect allows us to test for differences among extraction protocol and initial load (the main predictors of interest), while accounting for the fact that individual frogs were necessarily swabbed multiple times to collect samples for separate DNA extractions. We used the “MuMIn” package in R [60] to average the best-fit (within 2 AIC, Akaike Information Criterion) models and then used coefficients from the model averaging in a linear regression to predict the probability of Bd detection at varying levels of Bd load to compare PM and MN.

To determine if there was a relationship between live Bd load and post-preservation Bd load, we conducted two separate linear regressions (one each for PM and MN), using log_10_-transformed ZE data (all nonzero values). To compare results of Bd detection success and post-preservation ZE (log_10_-transformed) across all swab events and a subset of them (Events A-E, and A-C, respectively, Table 1), we used singlicate data in GLMMs (for Bd detection success) and a linear model (LM) (for post-preservation ZE). In the GLMMs and LM, we treated swab event as a fixed effect and the identity of each individual frog as a random effect and used likelihood ratio tests to evaluate the models with and without swab event included in the model. We conducted a post-hoc Tukey test on the LM to determine degree of difference across all singlicate (Events A-E) ZE results. All analyses were performed in the R programming environment [60].

## Results

### PM: extraction optimizations

None of the optimizations conducted on PM-extracted swabs increased Bd detection success or ZE values. Reducing the PM extract dilution from 1:10 to 1:5 (within Event D, Table 1) had no effect on ZE (t = -1.559, df = 8, p = 0.158) or Bd recovery rate (McNemar's Χ^2^ = 0.067, df = 1, p = 0.796). Within Event D, there was no significant difference in ZE values (t = -0.047, df = 4, p = 0.964) or recovery rate (McNemar's Χ^2^ = 0.111, df = 1, p = 0.739) when the Genereleaser was used. Within Event C, ZE was significantly reduced when extracts were treated with Genereleaser (t = -3.809, df = 9, p = 0.004); yet there was no significant difference in recovery rate with the treatment (McNemar's Χ^2^ = 0.067, df = 1, p = 0.796). When swabs were rehydrated with TE during the extraction step (Event C), neither ZE (t = 2.263, df = 5, p = 0.073) nor recovery rate were affected (McNemar's Χ^2^ = 0.727, df = 1, p = 0.394).

### DNA extraction comparison: PM and MN

**Fig 1:**
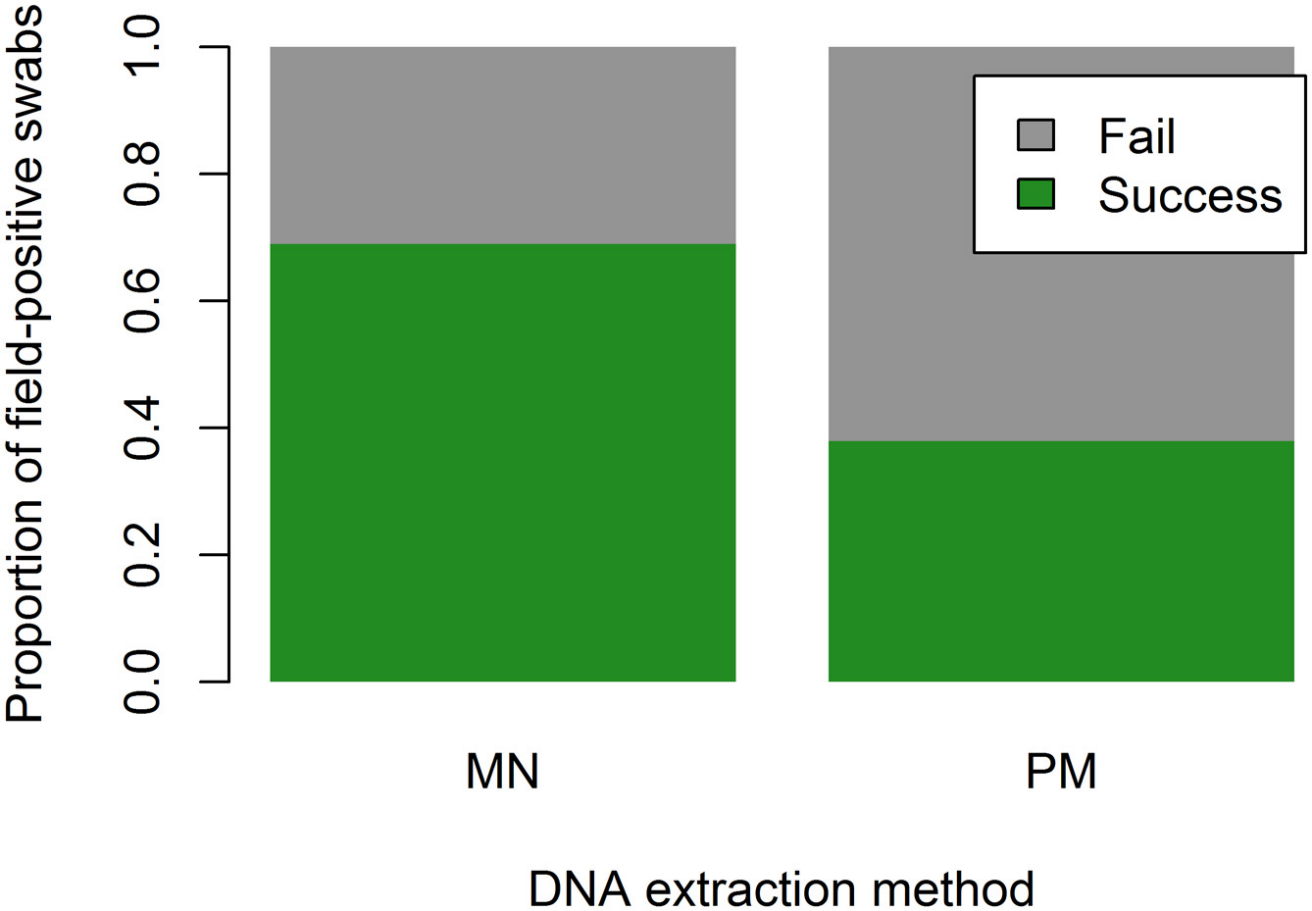
Comparison of overall Bd recovery success of Macherey-Nagel DNA FFPE extraction (MN) vs. PrepMan (PM). MN-extracted swabs were 31% more effective than PM at detecting Bd from formalin-fixed specimens that had been previously identified as Bd-positive in the field.

**Fig 2:**
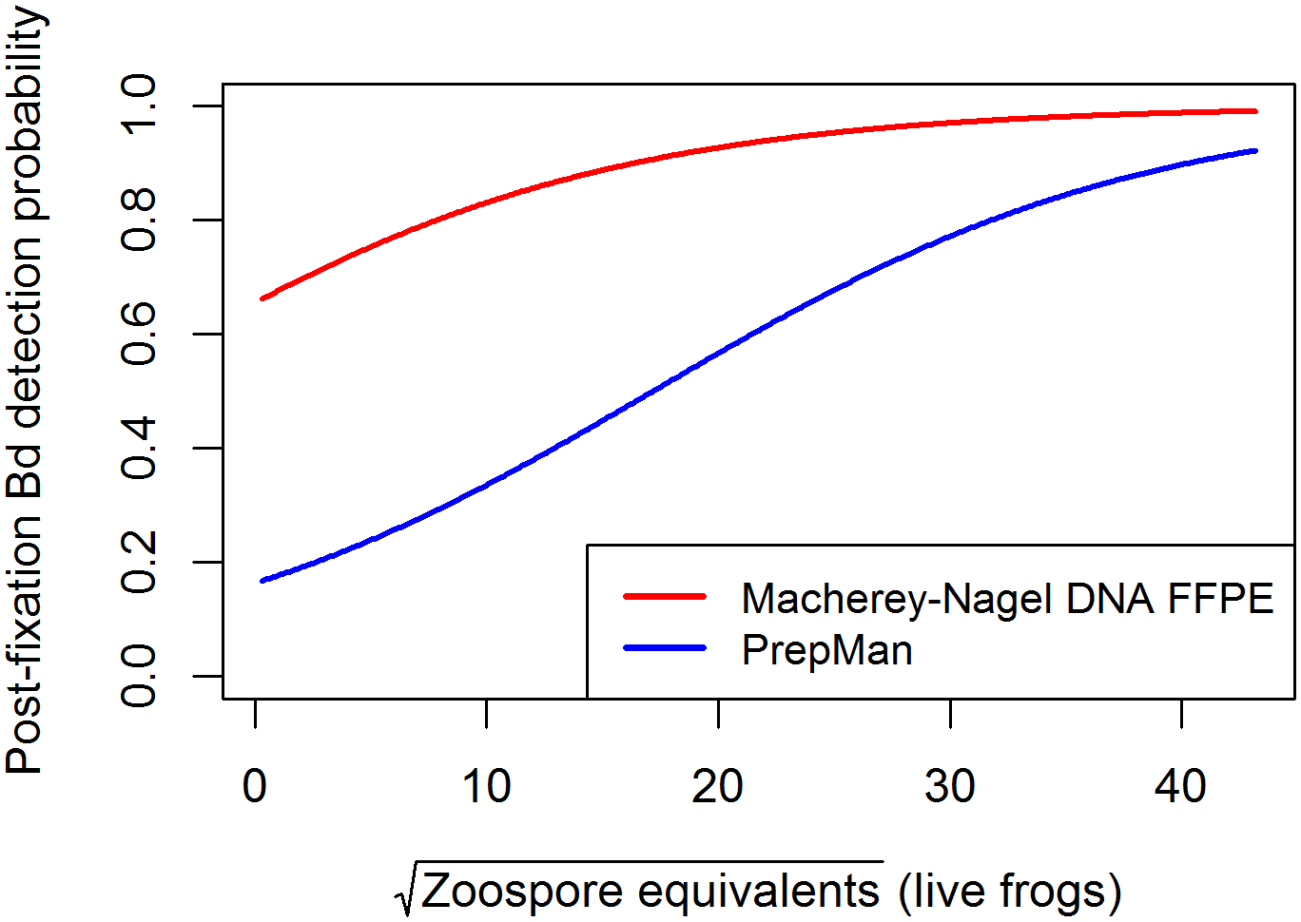
Probability of *Batrachochytrium dendrobatidis* (Bd) detection after formalin fixation. We used parameters from the best-fit generalized linear mixed models (Table 3) to predict Bd detection probabilities of the two different extraction methods, Macherey-Nagel DNA FFPE (MN), and PrepMan (PM). The differences in Bd detection probability between MN and PM are greatest at low and moderate Bd loads (as much as 50% at the median infection level of this study) and become more similar at higher Bd loads.

The MN extraction significantly increased the likelihood of Bd detection from formalin-fixed specimens over PM-extracted samples (McNemar's X^2^ = 13.5, df = 1, p = 0.0002, Fig 1), an overall increase in Bd detection of 31%. The regression analysis also indicated the importance of extraction method, and identified pre-preservation Bd load and individual frog mass as influencing Bd detection success, as evidenced by the best-fit models with the lowest AIC values (Tables 2 and 3). Based on the best-fit models, the probability of Bd detection is higher for MN extractions, with the greatest difference between PM and MN Bd detection success at low to moderate Bd loads; as loads get higher, the probabilities of Bd detection from MN- and PM-extracted swabs become more similar (Fig 2).

**Table 2:** Results of generalized linear models of post-preservation Bd detection success. All models included a random effect of individual frogs. The top three models included extract (PM and MN), pre-preservation Bd load (Live ZE), and individual frog mass.

Fixed effects	AIC	Δ AIC
Live ZE + Extract	127.2	
Live ZE * Extract	128.4	1.2
Live ZE + Extract + Frog Mass	128.5	1.3
Live ZE + Extract * Frog Mass	129.6	2.4
Live ZE * Extract + Frog Mass	129.8	2.6
Live ZE * Extract * Frog Mass	131.9	4.7
Extract	133.1	5.9
Extract + Frog Mass	134	6.8
Extract * Frog Mass	134.9	7.7
Live ZE	141.4	14.2
Live ZE * Frog Mass	142.1	14.9
Live ZE + Frog Mass	142.8	15.6
Frog Mass	147.9	20.7

**Table 3:** Results of model averaging for three best-fit (within 2 AIC) models for the dependence of frog parameters on the probability of Bd detection as response variable in generalized linear mixed models, (Table 2; n = 52 frogs, 104 samples). Individual frog ID was included as a random factor. Individuals without mass data available (n = 6) were excluded from the analysis.

Parameter(s)	Estimate	SE	Z	Pr(>|z|)
(Intercept)	0.513	0.651	0.780	0.435
Live ZE	0.082	0.045	1.810	0.070
Extract (PM)	-2.274	0.782	2.874	0.004**
Extract (PM) * Live ZE	0.013	0.038	0.351	0.725
Frog Mass	0.0004	0.002	0.319	0.750

**p<0.01

**Fig 3:**
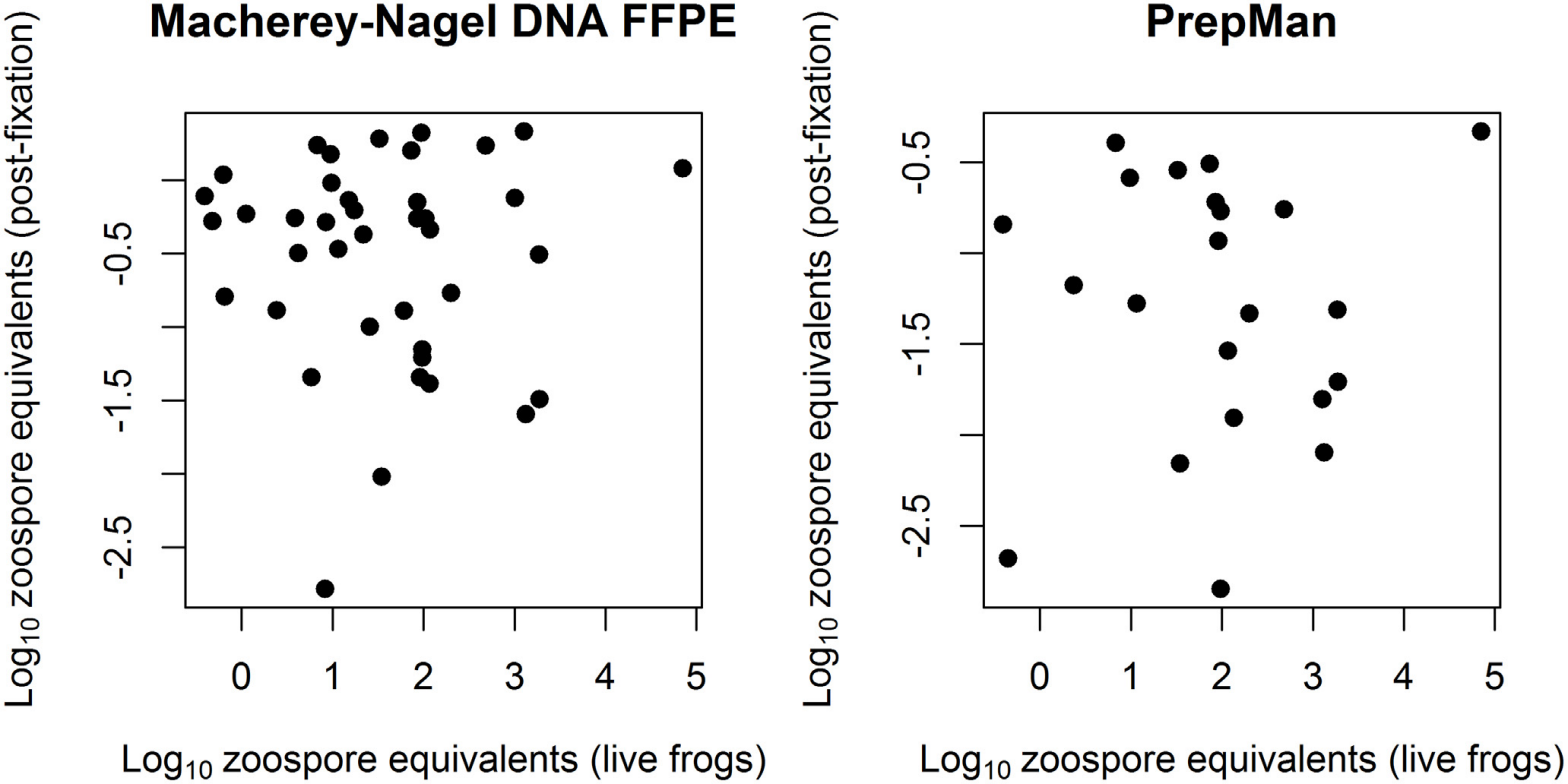
Nonzero zoospore equivalent values before and after formalin fixation for MN and PM-extracted swabs. There was no significant relationship between pre-and post-preservation Bd loads on individual swabs for either the MN (R^2^ = 0.0006; R^2^ (adj.) = -0.026; p = 0.879) or the PM (R^2^ = 0.003; R^2^ (adj.) = -0.046; p = 0.798) extracted specimen swabs.

There was no significant relationship between Bd loads (ZE) prior to and after formalin fixation for either the MN or the PM extractions (MN: F = 0.023, df = 38, p = 0.879; PM: F = 0.067, df = 20, p = 0.798; Fig 3), but pre-fixation Bd load was included in all of the best-fit GLMMs describing the probability of Bd detection, so its relevance cannot be completely discounted. The lowest ZE level on a live frog that was successfully detected after formalin fixation was 0.37.

**Fig 4:**
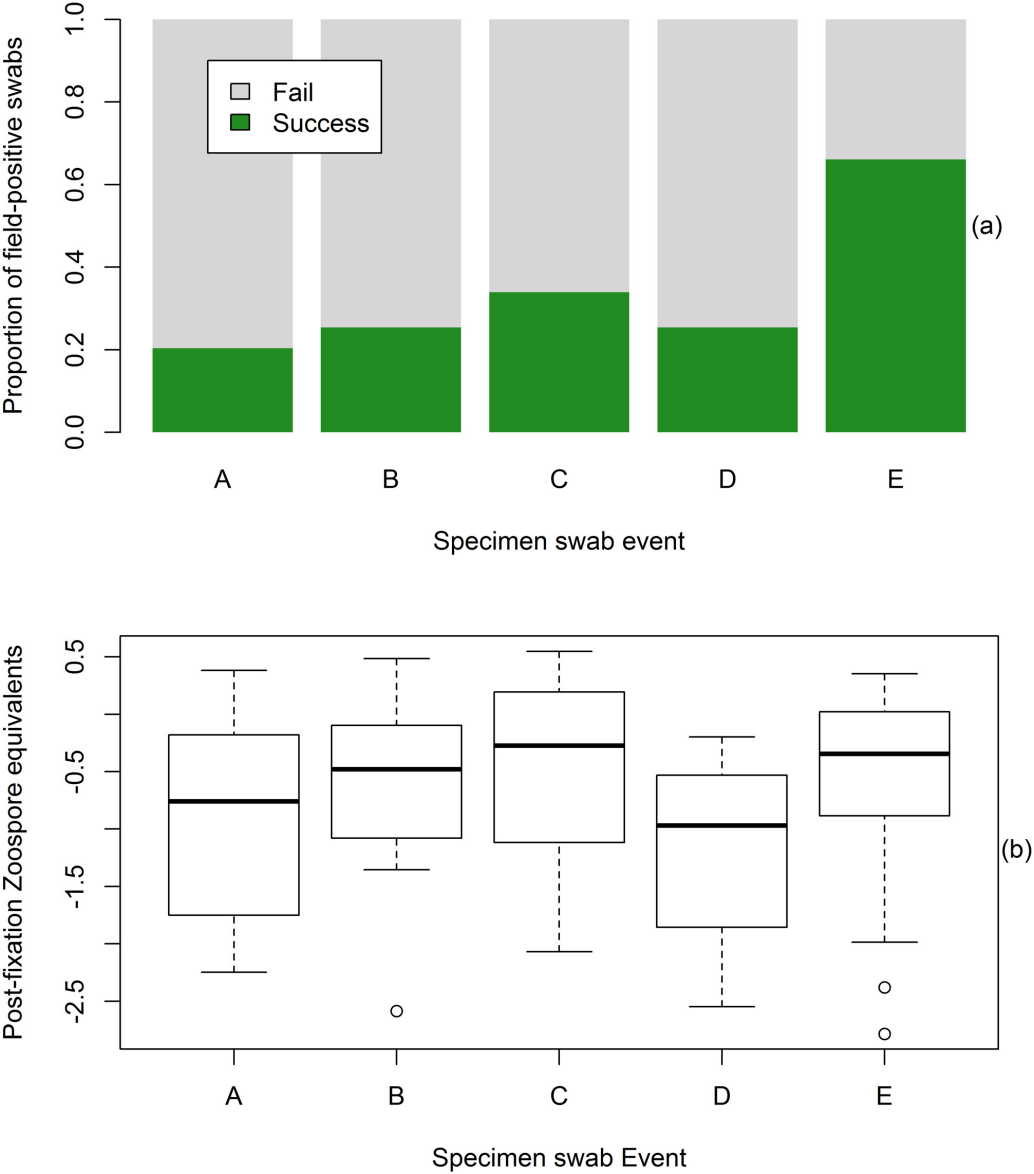
Post-fixation Bd detection success (top) and zoospore equivalents (bottom) across all swab events (n = 58). (a) Bd detection success was significantly greater for swab event E as compared to all other swab events (p < 0.0001). (b) The two ZE outliers in Event E are from specimens that had pre-preservation Bd loads of less than 1 zoospore equivalent and were not detected, in any number of runs, by any of the PM-extracted swabs. Results are based on analysis of singlicate data.

Increasing the number of swab strokes per frog (Events A-C, Table 1) increased Bd detection success within the PM-extracted swabs, though not significantly so (X^2^ = 2.90; df = 2; p = 0.235, Fig 4a). Post-preservation Bd detection success and ZE comparisons of singlicate results for all swab events (A through E) are shown in Fig 4. There was a significant effect of swab event on both Bd detection success (X^2^ = 41; df = 4; p < 0.0001, Fig 4a) and post-preservation ZE (X^2^ = 14.91; df = 4; p = 0.005, Fig 4b). Resulting post-preservation ZE across all PM-extracted swabs (Events A through D; nonzero values only) were not significantly different from each other, with the exception of Events C and D (Tukey’s HSD, p = 0.01). ZE from MN-extracted swabs (Event E) were significantly greater than the PM-extracted D event swabs (Tukey’s HSD, p = 0.003, Fig 4b). Bd detection did not necessarily increase successively with each swab event of equal swab strokes, as evidenced by the decrease in ZE and the proportion of Bd positive swabs detected (see Events C and D; Fig 4). This reduction between Events C and D could have been the result of reduced Bd DNA on the frogs as a consequence of previous swab events. None of the samples collected from the four Bd-negative control animals produced a (false) positive result in any of the replicates.

## Discussion

We compared two DNA extractions and various optimizations to improve Bd detection from formalin-fixed and ethanol-preserved frog skin. The greatest increase in Bd detection resulted from using the MN spin column-based DNA extraction kit. The MN extraction increased Bd detection by as much as 50% over PM-extracted samples, suggesting that its lack of inhibitors and increased quality of resultant DNA allows for increased DNA detection over PM. This is a similar result to other studies, in which samples extracted with spin column-based extraction kits such as Qiagen DNeasy and Qiagen Blood and Tissue increased Bd detection over PM by 7% and 23%, respectively [24, 49]. In the standard Bd assay using PM, the extract must be diluted 1:10 prior to qPCR because PM is a relatively rudimentary extraction method that allows proteins from the DNA into the extract, which inhibit PCR reactions [39, 61]. All of the optimizations of the PM protocol attempted in this study were unsuccessful. Our results support those of Cheng et al. [24]: PM-extracted swabs from formalin-fixed specimens with high pre-fixation fungal load can accurately detect Bd 80–90% of the time (Fig 2). However, as Bd loads decrease, Bd detection success also decreases, and considerably more so for PM-extracted swabs than for MN-extracted swabs (Fig 2). This is a critical consideration, since the large majority of amphibians in museums are not likely to have high Bd loads.

Although increased Bd load on live frogs increases the probability of detection after formalin fixation, there is likely not an identifiable pre-fixation load below which Bd cannot be detected post-fixation. The lowest Bd load from a live frog that was detected post-preservation was 0.37 ZE. The lack of a relationship between the Bd load of live animals and the Bd load detected after formalin fixation, as shown by this study (Fig 3) and others [24, 43] show that post-preservation ZE cannot be used to accurately infer pre-fixation Bd loads. Similarly, historical Bd prevalence should be deduced with caution, considering the relatively low Bd recovery rates for both the PM and MN extraction methods at low Bd loads (Fig 2). The percentage of Bd-positive specimens in a given sample cannot be considered an accurate measure of actual historical pathogen prevalence for most historical amphibian populations. Nonetheless, describing Bd prevalence through time can provide a relative measure of the pathogen’s incidence in the landscape [49], although we did not specifically test the impact of time since fixation on Bd detection. The multiple swab events conducted may have reduced the amount of Bd DNA on the frogs to be sampled in successive swab events, as indicated in the reduced ZE loads between the PM-extracted Events C and D (Fig 4). Consequently, the difference in Bd detection success between PM- and MN-extracted swabs is potentially much greater than shown by this study.

Incidence of false positives is an extremely important factor to consider when using qPCR to detect Bd in formalin-fixed museum specimens, due to the higher sensitivity of qPCR over standard end-point PCR. Here, four Bd-negative control animals that were stored with known Bd-positive animals produced negative runs through all of the replicates, showing that thorough rinsing with fresh ethanol prior to sampling may be an adequate method for reducing false positives from Bd-contaminated ethanol in shared storage jars. If careful sampling hygiene and adequate rinsing with fresh ethanol are conducted, and strict PCR laboratory decontamination protocols are adhered to, false positives in a sample can be avoided. We recognize that some may consider four negative controls out of 62 total animals a relatively small number on which to base this conclusion; however, it is important to note that these 4 negative control animals are represented by 28 individual qPCR runs that did not amplify. Not all preserved specimens in a single jar will be positive for Bd; therefore, these results show that mass contamination within a museum jar does not always happen [43].

Contemporary researchers examining historical Bd occurrences should be cognizant of the risks of relying solely on qPCR results for Bd detection and consider combining them with other methods (e.g., histology) in cases of equivocal or enigmatic results. Yet, because of the uneven distribution of chytridiomycosis on amphibian skin [39], histology is not a 100% accurate diagnostic, is laborious when attempting to assess large numbers of animals, and damages specimens. In at least one previous study, an animal that was confirmed Bd positive with PCR was determined negative by histology [46]. Given low detectability from specimens with low pre-fixation Bd loads (Fig 2), researchers should take replicates into account as compared to both positive and negative controls. The extremely small amount of intact Bd DNA remaining after formalin fixation increases the likelihood that each qPCR well will not receive a sample containing Bd. Instead of counting singlicate positives as negatives, researchers should consider re-running the extract and attempting detection again. In addition, the same specimen could be re-swabbed and re-analyzed. The reduction in detection between swab Events C and D in this study suggests that multiple swabbing events can reduce Bd DNA on frogs, although our results also show that when a spin column-based extraction is used, detection probability is relatively high even after frog skin has encountered multiple previous swabbing events.

Based on the results of this study, we make the following recommendations to maximize Bd DNA recovery rates from formalin-fixed museum specimens using qPCR: 1) Use MN or a similar spin-column based DNA extraction method, and/or one specifically designed for formalin-fixed sample types. Like previous research [24, 49, 61, 62], this study also finds that qPCR inhibition leads to under-estimates of Bd prevalence. The higher quality of MN-extracted DNA can facilitate future use of the samples in studies aimed at investigating Bd’s deeper molecular patterns, including genetics relating to strain differences, variations in virulence, and population genetics, in addition to allowing for increased Bd DNA detection using qPCR. We have estimated that switching from a PM to an MN extraction would cost approximately one dollar more per extraction, and both methods require equal amounts of labor; 2) When using a “clean” extraction method such as MN, use as much of the DNA extraction template as the master mix reaction volume allows to increase probability of Bd detection by replacing water in the reaction with template [43]; 3) Collect multiple swabs from the same animal so that equivocal results can be re-extracted and re-run to confirm equivocal positives [43]; 4) Run samples in duplicate, and if a sample amplifies in one of the duplicate reactions, run the sample a third time to determine if it should be considered positive or not. This recommendation is also supported by the work of Cheng et al. [24], which found that runs in triplicate or quadruplicate did not significantly differ in their ability to ascertain Bd infection status, as determined by histology, over duplicate runs of the same sample.

DNA detection from formalin-fixed tissues is challenging [63]. Establishing clear protocols for most effectively and reliably detecting Bd DNA with qPCR from formalin-fixed specimens will significantly reduce the number of specimens damaged by histological methods. These methods can be used to more accurately sample a large number of specimens and evaluate landscape-scale questions, such as the emergence and prevalence of Bd in threatened amphibian populations. Stemming amphibian declines and extinctions worldwide calls for an extraordinary, coordinated response effort [64]. Critical to guiding these efforts is establishing protocols that are widely used and agreed upon to ensure judicious and comparable deductions from historical Bd data.

## References

[1] Rocha L, Aleixo A, Allen G, Almeda F, Baldwin C, Barclay M, et al Specimen collection: an essential tool. Science (New York, NY). 2014;344(6186): 814. 10.1126/science.344.6186.814 24855245

[2] Lips KR. Museum collections: Mining the past to manage the future. Proc Natl Acad Sci USA. 2011;108(23): 9323–4. 10.1073/pnas.1107246108 21610166 PMC3111260

[3] Porter RD, Wiemeyer SN. Dieldrin and DDT: Effects on Sparrow Hawk Eggshells and Reproduction. Science. 1969;165(3889): 199–200. 10.1126/science.165.3889.199 5815079

[4] Gardner JL, Peters A, Kearney MR, Joseph L, Heinsohn R. Declining body size: a third universal response to warming? Trends Ecol Evol. 2011;26(6): 285–91. 10.1016/j.tree.2011.03.005 10.1016/j.tree.2011.03.005 21470708

[5] Avila-Arcos MC, Ho SYW, Ishida Y, Nikolaidis N, Tsangaras K, Honig K, et al One Hundred Twenty Years of Koala Retrovirus Evolution Determined from Museum Skins. Mol Biol Evol. 2013;30(2): 299–304. 10.1093/molbev/mss223. WOS:000314122000007 22983950 PMC3548305

[6] Wyatt KB, Campos PF, Gilbert MTP, Kolokotronis S-O, Hynes WH, DeSalle R, et al Historical Mammal Extinction on Christmas Island (Indian Ocean) Correlates with Introduced Infectious Disease. PLoS One. 2008;3(11): e3602 10.1371/journal.pone.0003602 18985148 PMC2572834

[7] Weldon C, Du Preez L, Hyatt AD, Muller R, Speare R. Origin of the amphibian chytrid fungus. Emerging Infect Dis. 2004;10(12): 2100–5. 15663845 10.3201/eid1012.030804 PMC3323396

[8] Hewson I, Button JB, Gudenkauf BM, Miner B, Newton AL, Gaydos JK, et al Densovirus associated with sea-star wasting disease and mass mortality. Proc Natl Acad Sci. 2014;111(48): 17278–83. 10.1073/pnas.1416625111 25404293 PMC4260605

[9] Berger L, Speare R, Daszak P, Green DE, Cunningham AA, Goggin CL, et al Chytridiomycosis causes amphibian mortality associated with population declines in the rain forests of Australia and Central America. Proc Natl Acad Sci. 1998;95(15): 9031–6. 9671799 10.1073/pnas.95.15.9031 PMC21197

[10] Pessier AP, Nichols DK, Longcore JE, Fuller MS. Cutaneous Chytridiomycosis in Poison Dart Frogs (*Dendrobates* spp.) and White's Tree Frogs (*Litoria Caerulea*). J Vet Diagn Invest. 1999;11(2): 194–9. 10098698 10.1177/104063879901100219

[11] Rachowicz LJ, Knapp RA, Morgan JAT, Stice MJ, Vredenburg VT, Parker JM, et al Emerging infectious disease as a proximate cause of amphibian mass mortality. Ecology. 2006;87(7): 1671–83. 16922318 10.1890/0012-9658(2006)87[1671:eidaap]2.0.co;2

[12] Muths E, Corn PS, Pessier AP, Green DE. Evidence for disease-related amphibian decline in Colorado. Biol Conserv. 2003;110(3): 357–65. 10.1016/s0006-3207(02)00239-2. WOS:000181189400005

[13] Heard M, Smith KF, Ripp K. Examining the evidence for chytridiomycosis in threatened amphibian species. PLoS One. 2011;6(8): e23150 10.1371/journal.pone.0023150 21826233 PMC3149636

[14] Olson DH, Aanensen DM, Ronnenberg KL, Powell CI, Walker SF, Bielby J, et al Mapping the global emergence of *Batrachochytrium dendrobatidis*, the amphibian chytrid fungus. PLoS One. 2013;8(2): e56802 10.1371/journal.pone.0056802 23463502 PMC3584086

[15] Skerratt LF, Berger L, Speare R, Cashins S, McDonald KR, Phillott AD, et al Spread of Chytridiomycosis Has Caused the Rapid Global Decline and Extinction of Frogs. EcoHealth. 2007;4(2): 125–34. 10.1007/s10393-007-0093-5

[16] Kilpatrick AM, Briggs CJ, Daszak P. The ecology and impact of chytridiomycosis: an emerging disease of amphibians. Trends Ecol Evol. 2010;25(2): 109–18. 10.1016/j.tree.2009.07.011. 19836101

[17] Morehouse EA, James TY, Ganley ARD, Vilgalys R, Berger L, Murphy PJ, et al Multilocus sequence typing suggests the chytrid pathogen of amphibians is a recently emerged clone. Mol Ecol. 2003;12(2): 395–403. 10.1046/j.1365-294X.2003.01732.x 12535090

[18] Morgan JA, Vredenburg VT, Rachowicz LJ, Knapp RA, Stice MJ, Tunstall T, et al Population genetics of the frog-killing fungus *Batrachochytrium dendrobatidis* . Proc Natl Acad Sci USA. 2007;104(34): 13845–50. 10.1073/pnas.0701838104 17693553 PMC1945010

[19] James TY, Litvintseva AP, Vilgalys R, Morgan JA, Taylor JW, Fisher MC, et al Rapid global expansion of the fungal disease chytridiomycosis into declining and healthy amphibian populations. PLoS Pathog. 2009;5(5): e1000458 10.1371/journal.ppat.1000458 19478871 PMC2680619

[20] Farrer RA, Weinert LA, Bielby J, Garner TW, Balloux F, Clare F, et al Multiple emergences of genetically diverse amphibian-infecting chytrids include a globalized hypervirulent recombinant lineage. Proc Natl Acad Sci USA. 2011;108(46): 18732–6. Epub 2011/11/09. 10.1073/pnas.1111915108 22065772 PMC3219125

[21] Lips KR, Diffendorfer J, Mendelson JR, Sears MW. Riding the wave: reconciling the roles of disease and climate change in amphibian declines. PLOS Biol. 2008;6(3): e72 10.1371/journal.pbio.0060072 18366257 PMC2270328

[22] Vredenburg VT, Knapp RA, Tunstall TS, Briggs CJ. Dynamics of an emerging disease drive large-scale amphibian population extinctions. Proc Natl Acad Sci USA. 2010;107(21): 9689–94. 10.1073/pnas.0914111107 20457913 PMC2906868

[23] Weinstein SB. An Aquatic Disease on a Terrestrial Salamander: Individual and Population Level Effects of the Amphibian Chytrid Fungus, *Batrachochytrium dendrobatidis*, on *Batrachoseps attenuatus* (Plethodontidae). Copeia. 2009;2009(4): 653–60.

[24] Cheng TL, Rovito SM, Wake DB, Vredenburg VT. Coincident mass extirpation of neotropical amphibians with the emergence of the infectious fungal pathogen *Batrachochytrium dendrobatidis* . Proc Natl Acad Sci USA. 2011;108(23): 9502–7. 10.1073/pnas.1105538108 21543713 PMC3111304

[25] Ouellet M, Mikaelian I, Pauli BD, Rodrigue J, Green DM. Historical Evidence of Widespread Chytrid Infection in North American Amphibian Populations. Conserv Biol. 2005;19(5): 1431–40. 10.1111/j.1523-1739.2005.00108.x

[26] Zhu W, Bai C, Wang S, Soto-Azat C, Li X, Liu X, et al Retrospective survey of museum specimens reveals historically widespread presence of *Batrachochytrium dendrobatidis* in China. EcoHealth. 2014: 1–10. 10.1007/s10393-013-0894-7 24419667

[27] Soto-Azat C, Clarke BT, Poynton JC, Cunningham AA. Widespread historical presence of *Batrachochytrium dendrobatidis* in African pipid frogs. Divers Distrib. 2010;16(1): 126–31. 10.1111/j.1472-4642.2009.00618.x

[28] de Queiroz Carnaval ACO, Puschendorf R, Peixoto OL, Verdade VK, Rodrigues MT. Amphibian chytrid fungus broadly distributed in the Brazilian Atlantic Rain Forest. EcoHealth. 2006;3(1): 41–8.

[29] Huss M, Huntley L, Vredenburg V, Johns J, Green S. Prevalence of *Batrachochytrium dendrobatidis* in 120 Archived Specimens of *Lithobates catesbeianus* (American Bullfrog) Collected in California, 1924–2007. EcoHealth. 2013;10(4): 339–43. 10.1007/s10393-013-0895-6 24419668

[30] Soto-Azat C, Valenzuela-Sanchez A, Clarke BT, Busse K, Ortiz JC, Barrientos C, et al Is Chytridiomycosis Driving Darwin's Frogs to Extinction? PLOS ONE. 2013;8(11). 10.1371/journal.pone.0079862. WOS:000327313100059 PMC3835940 24278196

[31] Berger L, Speare R, Kent A. Diagnosis of chytridiomycosis in amphibians by histologic examination. Zoos Print J. 1999;15: 184–90.

[32] Lips KR, Brem F, Brenes R, Reeve JD, Alford RA, Voyles J, et al Emerging infectious disease and the loss of biodiversity in a Neotropical amphibian community. Proc Natl Acad Sci USA. 2006;103(9): 3165–70. 10.1073/pnas.0506889103 16481617 PMC1413869

[33] Padgett-Flohr GE, Hopkins RL 2nd. *Batrachochytrium dendrobatidis*, a novel pathogen approaching endemism in central California. Dis Aquat Org. 2009;83(1): 1–9. 10.3354/dao02003 19301630

[34] Longcore JE, Pessier AP, Nichols DK. *Batrachochytrium dendrobatidis gen*. *et sp*. *nov*., a chytrid pathogenic to amphibians. Mycologia. 1999;91(2): 219–27.

[35] Berger L, Marantelli G, Skerratt LF, Speare R. Virulence of the amphibian chytrid fungus *Batrachochytium dendrobatidis* varies with the strain. Dis Aquat Org. 2005;68(1): 47–50. 10.3354/dao068047 16465833

[36] Padgett-Flohr GE. *Batrachochytrium dendrobatidis* in central California amphibians [Dissertation]: Southern Illinois University Carbondale; 2009.

[37] Reeder NMM, Pessier AP, Vredenburg VT. A reservoir species for the emerging amphibian pathogen *Batrachochytrium dendrobatidis* thrives in a landscape decimated by disease. PLoS One. 2012;7(3). 10.1371/journal.pone.0033567. WOS:000302381500155 PMC3299797 22428071

[38] Daszak P, Strieby A, Cunningham AA, Longcore JE, Brown CC, Porter D. Experimental evidence that the bullfrog (*Rana catesbeiana*) is a potential carrier of chytridiomycosis, an emerging fungal disease of amphibians. Herpetolog J. 2004;14(4): 201–7. WOS:000225639500006

[39] Boyle DG, Boyle DB, Olsen V, Morgan JAT, Hyatt AD. Rapid quantitative detection of chytridiomycosis (*Batrachochytrium dendrobatidis*) in amphibian samples using real-time Taqman PCR assay. Dis Aquat Organ. 2004;60(2): 141–8. 10.3354/dao060141. WOS:000224225800007 15460858

[40] Annis SL, Dastoor FP, Ziel HZ, Daszak P, Longcore JE. A DNA-based assay identifies *Batrachochytrium dendrobatidis* in amphibians. J Wildl Dis. 2004;40(3): 420–8. 15465708 10.7589/0090-3558-40.3.420

[41] Kriger KM, Hines HB, Hyatt AD, Boyle DG, Hero JM. Techniques for detecting chytridiomycosis in wild frogs: comparing histology with real-time Taqman PCR. Dis Aquat Org. 2006;71: 141–8. 16956061 10.3354/dao071141

[42] Hyatt AD, Boyle DG, Olsen V, Boyle DB, Berger L, Obendorf D, et al Diagnostic assays and sampling protocols for the detection of *Batrachochytrium dendrobatidis* . Dis Aquat Org. 2007;73(3): 175–92. 10.3354/dao073175. WOS:000245222100001 17330737

[43] Richards-Hrdlicka KL. Extracting the amphibian chytrid fungus from formalin-fixed specimens. Methods in Ecology and Evolution. 2012;3(5): 842–9. 10.1111/j.2041-210X.2012.00228.x

[44] Popa OP, Murariu D, Popa LO. Comparison of four DNA extraction methods from invasive freshwater bivalve species (Mollusca: Bivalvia) in Romanian fauna. Trav Mus Natl Hist Nat Grigore Antipa. 2007;6: 527–36.

[45] Vredenburg VT, Felt SA, Morgan EC, McNally SVG, Wilson S, Green SL. Prevalence of *Batrachochytrium dendrobatidis* in *Xenopus* Collected in Africa (1871–2000) and in California (2001–2010). PLOS ONE. 2013;8(5): e63791 10.1371/journal.pone.0063791 23691097 PMC3655066

[46] Mendelson JR III, Jones ME, Pessier A, Toledo G, Kabay EH, Campbell JA. On the Timing of an Epidemic of Amphibian Chytridiomycosis in the Highlands of Guatemala. South American Journal of Herpetology. 2014;9(2): 151–3.

[47] Muletz C, Caruso NM, Fleischer RC, McDiarmid RW, Lips KR. Unexpected rarity of the pathogen *Batrachochytrium dendrobatidis* in Appalachian Plethodon salamanders: 1957–2011. PLOS ONE. 2014;9(8): e103728 10.1371/journal.pone.0103728 25084159 PMC4118919

[48] Rodriguez D, Becker C, Pupin N, Haddad C, Zamudio K. Long-term endemism of two highly divergent lineages of the amphibian-killing fungus in the Atlantic Forest of Brazil. Mol Ecol. 2014;23(4): 774–87. 10.1111/mec.12615 24471406

[49] Talley BL, Muletz CR, Vredenburg VT, Fleischer RC, Lips KR. A century of *Batrachochytrium dendrobatidis* in Illinois amphibians (1888–1989). Biol Conserv. 2015;182: 254–61.

[50] Fong JJ, Cheng TL, Bataille A, Pessier AP, Waldman B, Vredenburg VT. Early 1900s Detection of *Batrachochytrium dendrobatidis* in Korean Amphibians. PLOS ONE. 2015;10(3): e0115656 10.1371/journal.pone.0115656 25738656 PMC4349589

[51] Kinney VC, Heemeyer JL, Pessier AP, Lannoo MJ. Seasonal pattern of *Batrachochytrium dendrobatidis* infection and mortality in *Lithobates areolatus*: Affirmation of Vredenburg's “10,000 zoospore rule”. PLOS ONE. 2011;6(3): e16708 10.1371/journal.pone.0016708 21423745 PMC3053364

[52] Briggs CJ, Knapp RA, Vredenburg VT. Enzootic and epizootic dynamics of the chytrid fungal pathogen of amphibians. Proc Natl Acad Sci USA. 2010;107(21): 9695–700. 10.1073/pnas.0912886107 20457916 PMC2906864

[53] Piovia-Scott J, Pope K, Joy Worth S, Rosenblum EB, Poorten T, Refsnider J, et al Correlates of virulence in a frog-killing fungal pathogen: evidence from a California amphibian decline. ISME J. 2014 10.1038/ismej.2014.241 PMC4478697 25514536

[54] Green DE, Converse KA, Schrader AK. Epizootiology of sixty-four amphibian morbidity and mortality events in the USA, 1996–2001 In: Gibbs EPJ, Bokma BH, editors. Domestic Animal/Wildlife Interface: Issue for Disease Control, Conservation, Sustainable Food Production, and Emerging Diseases. Annals of the New York Academy of Sciences. 969. New York: New York Academy of Sciences; 2002 p. 323–39. 10.1111/j.1749-6632.2002.tb04400.x 12381613

[55] Bucklin A, Allen LD. MtDNA sequencing from zooplankton after long-term preservation in buffered formalin. Mol Phylogen Evol. 2004;30(3): 879–82. 10.1016/j.ympev.2003.11.002 15012969

[56] McDiarmid RW. Preparing Amphibians as Scientific Specimens In: Heyer WR, editor. Measuring and Monitoring Biological Diversity: Standard Methods for Amphibians. Washington, DC: Smithsonian Institution Press; 1994 p. 289–97.

[57] Simmons JE. Herpetological Collecting and Collections Managment In: Moriarty JJ, editor. Herpetological Circular No31: Society for the Study of Amphibians and Reptiles; 2002 p. 153.

[58] Savage AE, Sredl MJ, Zamudio KR. Disease dynamics vary spatially and temporally in a North American amphibian. Biol Conserv. 2011;144(6): 1910–5. 10.1016/j.biocon.2011.03.018

[59] Barta JL, Monroe C, Teisberg JE, Winters M, Flanigan K, Kemp BM. One of the key characteristics of ancient DNA, low copy number, may be a product of its extraction. Journal of Archaeological Science. 2014;46: 281–9.

[60] R Core Team. R: a language and environment for statistical computing, reference index version 2.15. 1. R Foundation for Statistical Computing, Vienna, Austria. 2012. Available: https://www.r-project.org/.

[61] Kosch T, Summers K. Techniques for minimizing the effects of PCR inhibitors in the chytridiomycosis assay. Mol Ecol Resour. 2013;13(2): 230–6. 10.1111/1755-0998.12041 23241137

[62] Garland S, Baker A, Phillott AD, Skerratt LF. BSA reduces inhibition in a TaqMan assay for the detection of *Batrachochytrium dendrobatidis* . Dis Aquat Organ. 2010;92: 113–6. 10.3354/dao02053 21268973

[63] Wandeler P, Hoeck PE, Keller LF. Back to the future: museum specimens in population genetics. Trends Ecol Evol. 2007;22(12): 634–42. 17988758 10.1016/j.tree.2007.08.017

[64] Mendelson JR, Lips KR, Gagliardo RW, Rabb GB, Collins JP, Diffendorfer JE, et al Biodiversity—Confronting amphibian declines and extinctions. Science. 2006;313(5783): 48-. 10.1126/science.1128396. WOS:000238850200019 16825553

